# *KIR* content genotypes associate with carriage of hepatitis B surface antigen, e antigen and HBV viral load in Gambians

**DOI:** 10.1371/journal.pone.0188307

**Published:** 2017-11-17

**Authors:** Louis-Marie Yindom, Maimuna Mendy, Christopher Bodimeade, Caroline Chambion, Peter Aka, Hilton C. Whittle, Sarah L. Rowland-Jones, Robert Walton

**Affiliations:** 1 University of Oxford, Nuffield Department of Medicine, Oxford, United Kingdom; 2 Medical Research Council (UK), Fajara, The Gambia; 3 International Agency for Research on Cancer, Lyon, France; 4 Demographic and Health Surveys, ICF International, Rockville, Maryland United States of America; 5 London School of Hygiene and Tropical Medicine, London, United Kingdom; 6 Warwick Medical School, University of Warwick, Coventry, United Kingdom; 7 Centre for Primary Care and Public Health, Barts and the London School of Medicine and Dentistry, Queen Mary University, London, United Kingdom; Centre de Recherche en Cancerologie de Lyon, FRANCE

## Abstract

**Background:**

Hepatocellular carcinoma (HCC) causes over 800,000 deaths worldwide annually, mainly in low income countries, and incidence is rising rapidly in the developed world with the spread of hepatitis B (HBV) and C (HCV) viruses. Natural Killer (NK) cells protect against viral infections and tumours by killing abnormal cells recognised by Killer-cell Immunoglobulin-like Receptors (KIR). Thus genes and haplotypes encoding these receptors may be important in determining both outcome of initial hepatitis infection and subsequent chronic liver disease and tumour formation. HBV is highly prevalent in The Gambia and the commonest cause of liver disease. The Gambia Liver Cancer Study was a matched case-control study conducted between September 1997 and January 2001 where cases with liver disease were identified in three tertiary referral hospitals and matched with out-patient controls with no clinical evidence of liver disease.

**Methods:**

We typed 15 *KIR* genes using the polymerase chain reaction with sequence specific primers (PCR-SSP) in 279 adult Gambians, 136 with liver disease (HCC or Cirrhosis) and 143 matched controls. We investigated effects of *KIR* genotypes and haplotypes on HBV infection and associations with cirrhosis and HCC.

**Results:**

Homozygosity for *KIR* group A gene-content haplotype was associated with HBsAg carriage (OR 3.7, 95% CI 1.4–10.0) whilst telomeric A genotype (t-AA) was associated with reduced risk of e antigenaemia (OR 0.2, 95% CI 0.0–0.6) and lower viral loads (mean log viral load 5.2 vs. 6.9, p_c_ = 0.022). One novel telomeric B genotype (t-ABx2) containing *KIR3DS1* (which is rare in West Africa) was also linked to e antigenaemia (OR 8.8, 95% CI 1.3–60.5). There were no associations with cirrhosis or HCC.

**Conclusion:**

Certain *KIR* profiles may promote clearance of hepatitis B surface antigen whilst others predispose to e antigen carriage and high viral load. Larger studies are necessary to quantify the effects of individual *KIR* genes, haplotypes and *KIR/HLA* combinations on long-term viral carriage and risk of liver cancer. *KIR* status could potentially inform antiviral therapy and identify those at increased risk of complications for enhanced surveillance.

## Introduction

Hepatocellular carcinoma (HCC) is the commonest form of primary liver cancer and responsible for more than 800,000 deaths annually worldwide [1]. Developing countries carry the highest burden of disease with 70–85% of affected people living in Sub-Saharan Africa and Asia [2]. In The Gambia, HCC is the commonest cancer in men [3].

Hepatitis B (HBV) and C (HCV) viruses are both major risk factors for liver cirrhosis and HCC, together with exposure to aflatoxin in the diet which is common in West Africa [4,5,6]. Epidemiological studies show that when chronic viral hepatitis and dietary aflatoxin are both present, the increase in risk of HCC is at least multiplicative [7,8]. HBV and HCV infections are endemic in The Gambia, affecting over 90% of the population, with 15% to 20% chronically infected with hepatitis B and 3% hepatitis C [4,9,10]. Detectable HBV viraemia confers a six fold increase in risk of HCC, and higher levels (more than 10,000 viral copies/mL in serum) are strongly associated with both HCC and cirrhosis (17- and 39-fold increased risk respectively) [6].

NK cells play an important role in the innate host defence against viral infection and tumour transformation and thus could be important in protecting against chronic hepatitis infection and HCC. The actions of NK cells are mediated by direct cytotoxicity and secretion of cytokines [11]. Cytotoxicity is controlled by tightly balanced opposing signals from inhibitory and activatory killer-cell immunoglobulin-like receptors (KIR) present on the NK cell surface. Cytokine secretion mediates interaction between NK cells and agents of the adaptive immune system such as dendritic cells and T lymphocytes to modulate the host immune response to pathogens and to abnormal cells [12].

The key function of KIR receptors is to survey the surfaces of other (target) cells to detect the presence or absence of their cognate ligands which are human leukocyte antigen (HLA) class I molecules. KIR and HLA interact in a complex fashion which may result in target cell death [13,14]. Both activating and inhibitory pathways are likely to be governed by signals transduced from interactions between specific KIR and HLA molecules. The control of the system is such that reduction in MHC expression, as is often observed in tumours and virally infected cells, increases the likelihood of killing by NK cells because of lack of *KIR* inhibition. This ‘missing self’ model [15] proposed for NK cell detection of down regulation of *HLA* class I makes *KIR* genotype important in a wide range of human diseases [16].

*HLA* class I down regulation by hepatitis B virus has been demonstrated in hepatoblastoma cell lines [17] and decreased NK cell activity is also seen in patients with liver cancer [18]. In addition, the degree of reduction in NK cell function may be associated with increased risk of more invasive disease [19]. Several studies have investigated the role of *KIR* genes in a range of virus related cancers [20,21,22,23] and suggested that presence of activating *KIR* confers increased cancer risk–particularly for nasopharyngeal carcinoma [20] and cervical cancer [21].

Expression of activating KIR molecules is associated with susceptibility to chronic HCV infection and cirrhosis and with inability to clear HCV following initial infection [22]. The compound genotype *KIR3DS1/Bw4-80I* that favours NK cell activation is associated with chronic HCV carriage in people with hepatocellular carcinoma [23] and inactivating *KIR3DL1* is associated with spontaneous HCV clearance [24]. Whilst studies in hepatitis B are less common there is a suggestion that inactivating KIR may also protect against development of chronic HBV infection [25].

In contrast however in other viral infections, activating *KIR* may be advantageous to the host; for example KIR3DS1-HLA Bw4-80I confers protection from rapid progression to AIDS following HIV-1 infection although the underlying mechanisms are likely to be different [26]. Some reports suggest that interaction between KIR molecules and their ligands on the target cell surface is peptide specific [27,28] although this may not be universal [29].

**Fig 1:**
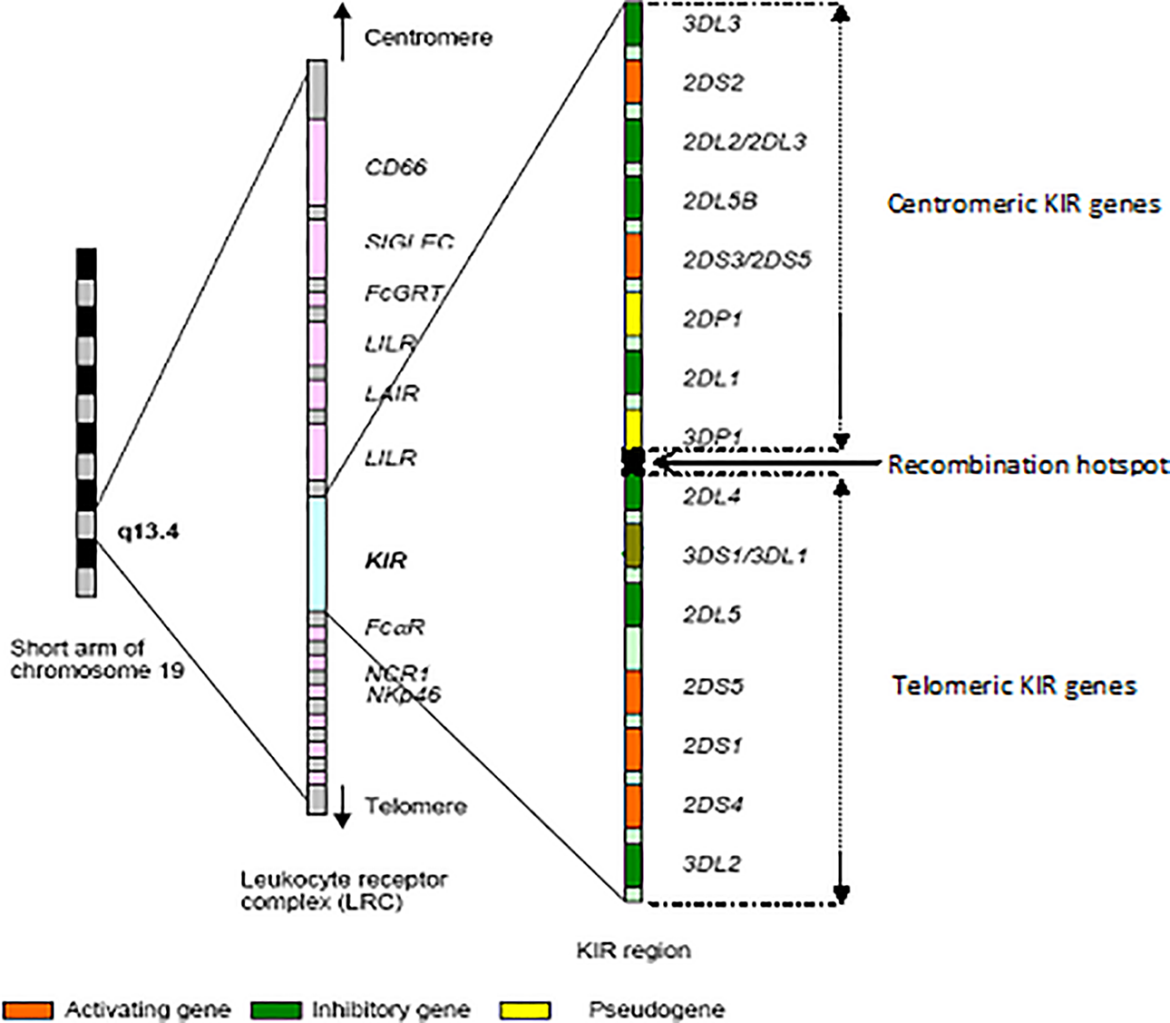
The genomic organisation of *KIR* genes on human chromosome 19. *KIR* genes are tightly organised head-to-tail over approximately 150 kb within the Leukocyte Receptor Complex (LRC). Inhibitory *KIR* genes are shown in green, activating genes in orange, pseudogenes in yellow, and the recombination hotspot in black. *KIR* genes vary in size ranging from 10 kb to 16 kb and are separated from each other by about 2 kb of intergenic space, except for the 14 kb recombination hotspot zone upstream of *KIR2DL4* that separates telomeric from centromeric KIRs.

KIR molecules in humans are encoded by a family of 16 genes located on chromosome 19 (Fig 1). This region of the human genome (approximately 150kb within the leukocyte receptor complex [LRC]) is highly polymorphic and its organisation has yet to be mapped fully in worldwide populations.

At the genomic level, *KIR* genes segregate into two clusters around a recombination hotspot which are designated ‘centromeric’ and ‘telomeric’, each of which contains a variable number of *KIR* genes. The composition and distribution of these clusters in populations distinguish individuals and ethnic groups. Studies on differences in *KIR* gene profiles (combinations of the centromeric and telomeric elements of the KIR locus) between populations suggest a differential contribution of each cluster to disease outcome [30,31]. Recent epidemiological evidence has implicated telomeric B haplotypes containing activating genes with slow progression of chronic hepatitis C infection in American Aboriginals [32,33].

Whilst there is accumulating evidence for an important role for KIR in hepatitis C infection [34], progression of disease [22], and development of hepatocellular carcinoma [23], there have been few studies investigating KIR associations in HBV infection and subsequent liver disease and cancer [35,36], although the underlying pathophysiological processes are likely to be similar. In addition the full range of the *KIR* gene repertoire has not been fully mapped in the genetically highly diverse African populations where hepatitis B is endemic and gives rise to high rates of chronic hepatitis and hepatocellular carcinoma.

Thus in the present study, we characterised KIR profiles and studied the effects of centromeric and telomeric gene clusters on immunological markers of hepatitis B infection, viral load, and liver disease in a well-characterised study group established by the International Agency for Research on Cancer (IARC) Gambia Hepatitis Intervention Study (GHIS) program under the Gambia Liver Cancer Case control study (GLCS) [4,6,37] in collaboration with the UK Medical Research Council Laboratories in The Gambia and the Gambian Government.

## Materials and methods

### Study population

Participants were adult Gambians recruited into the GLCS between September 1997 and January 2001. DNA for the current study was available from 279 of 624 original participants (Table 1).

**Table 1:** Characteristics of study participants.

	HCC N (%)	Cirrhosis N (%)	Liver disease N (%)	Control N (%)	OR
Total number	94	42	136	143	
**Gender**					
Male	76 (80.8)	30 (71.4)	106 (77.9)	94 (65.7)	NA
**Hepatitis B surface antigenaemia**					
Number tested	86	41	127	129	7.13
HBsAg+	54 (62.8)	29 (70.7)	83 (65.3)	27 (20.9)	
**Hepatitis B "e" antigenaemia**					
Number tested	38	21	59	12	NA
HBeAg+	8 (21.1)	8 (38.1)	16 (27.1)	0	
**HCV infection**					
Number tested	90	42	132	142	2
HCVAb	13 (14.4)	6 (14.3)	19 (14.4)	11 (7.8)	
**HBV viral load**					
Number tested	38	21	59	12	NA
MeanLog_10_HBV VL*	6.0 (4.5–7.3)	6.2 (4.5–7.6)	6.1 (4.5–7.6)	2.8 (2.4–3.1)	

*: mean log HBV viral load (VL quantified as copies per mL) and inter-quartile range

N: number tested, NA: not applicable, liver disease: HCC+ Cirrhosis

The GLCS was a case–control study conducted at three tertiary referral hospital sites in The Gambia to determine the burden of hepatocellular carcinoma in the entire nation and to examine aetiological factors [38]. Inclusion and exclusion criteria and methods have been described in detail elsewhere [4,6,37]. Briefly, patients with cancer and cirrhosis were identified from liver disease referral clinics at each of three sites (Royal Victoria Teaching Hospital (RVTH), the Medical Research Council Clinic in Fajara and Bansang Hospital). Diagnosis of primary liver cancer was confirmed by a) clinical evaluation b) serum alpha-fetoprotein levels >100 ng/mL c) compatible ultrasound findings and/or histopathology diagnosis. A subset of the cases had the diagnosis confirmed by liver biopsy (n = 54). Controls without clinical evidence of liver disease were recruited from the outpatient clinics of the same hospitals, frequency matched by sex and age (within 10 year groupings), and had normal alpha fetoprotein levels.

The Gambian population is ethnically highly diverse with five common culturally and linguistically distinct groups. Participant evaluation included a structured interview that recorded reported ethnicity, sociodemographic, lifestyle, and dietary factors. Blood and urine samples were also collected together with extensive information from a standardized clinical examination. DNA was extracted from blood samples and archived at -80 ^o^C.

### *KIR* typing

Genomic DNA was typed for 14 functional *KIR* genes: *2DL1-5*; *2DS1-5*; *3DL1-3*; *3DS1* and one pseudogene (*KIR2DP1*) using the polymerase-chain reaction with sequence-specific primers (PCR-SSP) [39]. Two pairs of KIR-specific primers were used to amplify segments of different sizes from the same *KIR* gene if present. PCR products were stained with ethidium bromide in 2% agarose gel and visualised on a UV light box. Gel pictures were independently scored for presence or absence of specific bands by two experienced scientists. Samples with discrepant results were repeated and the gene considered present if one of the reaction pairs was consistently positive upon repeat.

Mutations at the primer binding sites were confirmed by bi-directional sequencing using primers flanking the region under investigation. Briefly, primers were designed using known sequence from the KIR database [40]. The primers were then used to amplify a segment including the binding sites where the SSP primer under investigation was designed to anneal. The PCR product was then sequenced in both directions and traces analysed for mutations using the Mutation Surveyor software (SoftGenetics LLC., State College, PA 16803, USA).

The use of two pairs of primers in the PCR-SSP technique to detect the same gene was to limit false negative results due to PCR failure. The absence of specific bands on both reactions was confirmed by repeating the typing and the gene was only considered absent when both reactions remained negative following repeat. PCR efficiency was quality-controlled by adding a pair of primers to every reaction which amplified a 796bp conserved fragment from the third intron of the HLA-DRB1 gene.

### Analysis of *KIR* gene motifs

*KIR* genes were assigned to centromeric and telomeric loci based on their chromosomal positions relative to the recombination hotspot (RS) located between the framework genes *KIR3DP1* and *KIR2DL4*. *KIR* genes located upstream of RS are in the centromeric (Cen) gene cluster whereas those located downstream are telomeric (Tel). The methods were derived from techniques described by Cooley et al. [41] and Pyo et al. [42]. The gene-content of each of these loci (Cen or Tel) per chromosomal strand is known as “motif” which carries a variable number of activating and inhibitory *KIR* genes. Thus each sample has two Cen and two Tel motifs making its centromeric genotype (e.g. c-AA or c-AB) and telomeric genotype (e.g. t-AA or t-AB). The gene-content haplotype (also known as KIR profile) of each participant, spanning the recombination hotspot, was constructed by combining each Cen locus to its corresponding Tel locus. The centromeric part of the locus may contains up to 10 *KIR* genes including *KIR3DL3*, *2DS2*, *2DL2*, *2DL3*, *2DL5*, *2DS3*, *2DS5*, *2DP1*, *2DL1* and *3DP1*. The remainder (*KIR2DL4*, *3DL1*, *3DS1*, *2DS1*, *2DS4*, and *3DL2*) are located in the telomeric cluster.

*KIR* haplotypes are classified as group A or B depending on the presence of specific genes. Thus group A haplotypes contain the inhibitory genes *KIR3DL3*, *2DL3*, *2DL1*, *2DL4*, *3DL1*, and *3DL2*, together with a single activating gene *KIR2DS4*. In contrast, group B haplotypes are characterized by the presence of more than one activating *KIR* genes [43].

### Data analysis

All statistical analyses were performed using STATA version 9.2 (Stata Corporation, Texas, USA). Frequencies of the various genotypes and gene-content haplotypes were determined and compared between groups. Observed *KIR* gene frequencies were determined by direct counting and compared between HCC, Cirrhosis and Control groups using the Chi-square or Fisher exact tests as appropriate. Viral load measurements were log transformed to normalise the distribution and then compared between groups using t-tests. All p-values were corrected for multiple comparisons using the Bonferroni technique.

### Ethics statement

All participants were adults and gave written informed consent [4]. The GLCS was approved by the Gambia Government/MRC Joint Ethics Committee (SCC 648).

## Results

### Patient characteristics

Approximately half of the participants had liver disease—either HCC or cirrhosis (Table 1). Those with liver disease were more likely than controls to be hepatitis B surface antigen positive. A fifth of control participants were long-term HBV carriers (HBsAg+). There were no significant differences in *KIR* gene frequencies or HBsAg carriage between ethnic groups. The prevalence of risk factors and associations with disease status were similar to the original study [4,6].

### *KIR* telomeric AA genotype is associated with absence of hepatitis B e antigen and lower viral loads

**Fig 2:**
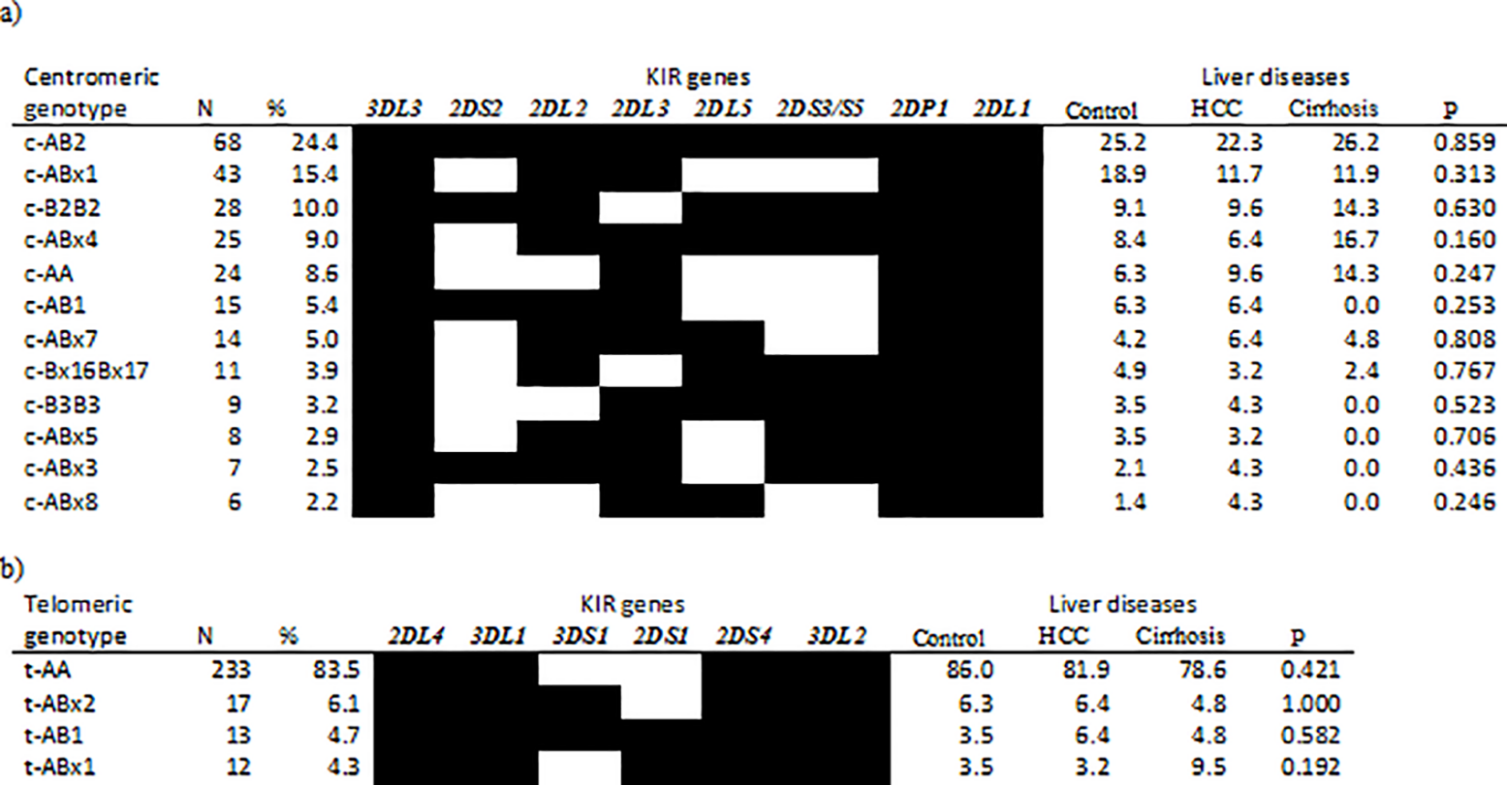
Centromeric and telomeric genotype distribution. a) Genomic organisation of centromeric genotypes present in at least 2% of the study population, b) *KIR* genes present telomeric genotypes (those found in at least 2% of the study population). Filled box: gene is present; open box: gene is absent, x: known or novel motifs (e.g. Bx2: second novel B motif identified for the first time in this study), N: number of individuals carrying the genotype of interest.

We initially compared markers of long-term carriage of hepatitis B in participants with or without individual *KIR* genes (Table 2). Carriers of *KIR3DS1* were more likely to be HBeAg positive, although the gene was uncommon in this population. More detailed analysis of the *KIR* motifs showed that homozygosity for group A *KIR* genes at the telomeric part of the *KIR* locus (t-AA) was inversely related to hepatitis B e antigenaemia (Table 3). Carriers of this genotype also had significantly lower HBV viral loads. We observed the opposite effect with one of the novel telomeric genotypes which is heterozygous for A and B motifs at the telomeric cluster (t-ABx2). The single difference between t-ABx2 and t-AA is the presence of the activating gene *KIR3DS1* (Fig 2B). Participants with t-ABx2 genotypes were more likely than those lacking this genotype to be HBeAg-positive (Table 3).

**Table 2:** Effects of *KIR* genes on immune markers of hepatitis B infection and hepatitis B viral load.

*KIR*	HBsAg- N (%)	HBsAg+ N (%)	OR	95% CI	HBeAg- N (%)	HBeAg+ N (%)	OR	95% CI	Mean Log_10_HBV virus load				
									Absent		Present		P
*2DL2*	123(84)	88(80)	0.8	0.4–1.4	43(78)	14(88)	2.0	0.4–10.0		4.7	5.7	0.912	
*2DL3*	122(84)	90(82)	0.9	0.5–1.7	45(82)	11(69)	0.5	0.1–1.8		6.7	5.2	0.160	
*2DL5*	92(63)	64(58)	0.8	0.5–1.4	33(60)	11(69)	1.5	0.4–4.9		5.2	5.7	1.000	
*2DS1*	12(8)	12(11)	1.4	0.6–3.2	4(7)	2(13)	1.8	0.3–11.2		5.4	6.9	0.936	
*2DS2*	70(48)	50(46)	0.9	0.6–1.5	24(44)	11(69)	2.8	0.8–9.6		5.4	5.6	1.000	
*2DS3*	68(47)	40(36)	0.7	0.4–1.1	20(36)	8(50)	1.8	0.6–5.5		5.5	5.6	1.000	
*2DS5*	38(26)	36(33)	1.4	0.8–2.4	20(36)	6(38)	1.1	0.3–3.4		5.3	5.9	1.000	
***3DS1***	14(10)	12(11)	1.2	0.5–2.6	2(4)	6(38)	**15.9**	2.3–112.5		5.3	7.2	0.160	

HBsAg: hepatitis B surface antigen, HBeAg: hepatitis B e antigen; N: number of individuals carrying the gene of interest. 110 individuals were HBsAg+, 146 were HBsAg-; 16 individuals were e antigen positive and 55 were e antigen negative; Present: mean LogHBV virus load in those carrying the gene of interest; Absent: mean LogHBV virus load in those without the gene of interest; OR: Odd ratio; CI: confidence interval; P: p-values corrected for multiple comparison by the Bonferroni method; Bold: significant association.

**Table 3:** *KIR* genotypes and outcomes of hepatitis B infection.

*KIR*	HBsAg-	HBsAg+	OR	95% CI	HBeAg-	HBeAg+	OR	95% CI	Mean Log_10_HBV virus load		
	N (%)	N (%)			N (%)	N (%)			Absent	Present	P
**Cen genotype**											
c-AA	6(4)	16(15)	**4.0**	1.5–10.8	8(15)	1(6)	0.4	0.0–3.5	5.7	4.2	0.147
c-ABx3	2(1)	5(5)	3.4	0.7–18.2	2(4)	0(0)	n.a.	n.a.	5.6	4.7	1.000
**Tel genotype**											
t-AA	125(86)	89(81)	0.7	0.4–1.4	49(89)	9(56)	**0.2**	0.0–0.6	6.9	5.2	**0.022**
t-ABx2	8(6)	6(6)	1.0	0.3–3.0	2(4)	4(25)	**8.8**	1.3–60.5	5.4	6.6	0.400
**Profile**											
c-AA/t-AA	6(4)	15(14)	**3.7**	1.4–10.0	8(15)	1(6)	0.4	0.0–3.5	5.7	4.2	0.098
c-AB2/t-AA	24(16)	16(15)	0.9	0.4–1.7	9(16)	2(13)	0.7	0.1–3.8	5.7	4.5	0.156
**Haplotype**											
AA	6(4)	15(14)	**3.7**	1.4–10.0	8(15)	1(6)	0.4	0.0–3.5	5.7	4.2	0.147
AB	140(96)	92(84)	**0.2**	0.1–0.6	47(86)	14(88)	1.2	0.2–6.4	4.7	5.7	0.346
BB	0(0)	3(3)	n.a.	n.a.	0(0)	1(6)	n.a.	n.a.	5.5.	8.8.	n.a.
**B content**											
AB1	92(63)	62(56)	0.8	0.5–1.3	32(58)	6(38)	0.4	0.1–1.4	6.0	5.1	0.318
AB2	44(30)	23(21)	0.6	0.3–1.1	13(24)	6(38)	1.9	0.6–6.5	5.2	6.6	**0.045**
AB3	4(3)	7(6)	2.4	0.7–8.5)	2(4)	2(13)	3.8	0.5–30.6	5.5	6.5	1.000

HBsAg: hepatitis B surface antigen, HBeAg: hepatitis B e antigen; N: number of individuals carrying the gene of interest; 110 people were HBsAg+, 16 were e antigen positive. Present: mean LogHBV virus load in those carrying the genotype of interest, Absent: mean LogHBV virus load in those without the genotype of interest, OR: Odd ratio, CI: confidence interval, P: p-values Bonferroni corrected for multiple testing, n.a.: not applicable. Bold: P<0.05. B content: 1, 2 and 3 represent the number of B motifs per profile, x: known or novel motifs (e.g. Bx2: second novel B motif identified for the first time in this study).

### Heterozygosity for *KIR* content-haplotype is associated with reduced risk of hepatitis B surface antigen carriage

45 distinct *KIR* profiles (S1 Table) were present in 279 participants from a recombination of 27 Cen and 8 Tel genotypes (S1 Fig). The most frequent profiles were c-AB2/t-AA and c-ABx1/t-AA present in >15% of the study population (S1 Table).

Participants homozygous for group A haplotype (based on gene-content) were more likely to be HBsAg-positive (Table 3). Conversely, heterozygosity at the *KIR* locus level (i.e. carriage of both A and B haplotypes) was negatively related to HBsAg carriage. Homozygosity for group B haplotype was uncommon in this study group (Table 3 and S1 Table). Centromeric and telomeric KIR genotypes were unrelated to disease status (Fig 2).

## Discussion

We found that the *KIR* telomeric AA genotype which is linked to NK cell inhibition was associated with reduced risk of hepatitis B e antigen carriage. This antigen is a marker of persistent viral replication and accordingly participants with t-AA genotype had significantly lower viral loads. Conversely carriers homozygous for genes belonging to the group A haplotype at both centromeric and telomeric clusters were more likely to be HBsAg-positive. Thus whilst certain KIR profiles may protect from high levels of viraemia in established infection, NK cell activation appears to be important in influencing the outcome of infection with the hepatitis B virus.

### Effects of individual *KIR* genes on hepatitis B infection

Activating *KIR* genes are relatively infrequent in Africans compared to other populations worldwide. The low frequency of *KIR3DS1* that we observed agrees with published *KIR* gene frequency data from Sub-Saharan Africa [44,45,46]. We found that carriers of *KIR3DS1* were more likely to be HBeAg positive and to have high viral loads, although our estimates of the size of the effect are imprecise because of the low gene frequency and small sample sizes.

Whilst participants with the inhibitory *KIR2DL3* gene had lower virus loads these differences were not significant in our study. Previous reports have suggested an association between *KIR2DL3* and clearance of hepatitis C virus with subsequent protection from chronic hepatitis, and liver damage [47,48]. These associations are strongest when *KIR2DL3* is present together with its ligand *HLA-C* group 1. Recent studies showed that HLA-C1 homozygosity either individually or in combination with *KIR2DL3* is associated with outcomes of HBV infection such as chronic HBV carriage and HCC [36,47]. Further studies are needed either to establish an independent effect of *KIR2DL3* in hepatitis B infection or to confirm the protective effect of *KIR2DL3/HLA-C* combinations.

### Centromeric and telomeric haplotypes and KIR profiles

More than 80% of centromeric and telomeric genotypes and KIR profiles in this study were previously unreported and are likely to be specific to West Africa. The genotype c-AA is the most common centromeric genotype observed worldwide [31,33] although we found it to be less frequent in Gambians. The only activating gene on this genotype is *KIR2DS4* which is sometimes not expressed [49,50].

At the telomeric cluster t-AA genotypes predominate, as has been observed in studies on previous Gambian and other West African populations [51]. We discovered previously undescribed B motifs one of which (t-Bx2) differs from the common telomeric A motif (t-A) only by the presence of *KIR3DS1*. Carriers of both of these motifs (t-ABx2 genotype) were more likely to be HBeAg-positive with higher HBV viral loads than those negative for this genotype.

In all, 45 different KIR profiles were found in 279 participants, the most common of which resulted from a recombination of genes belonging to group A and B haplotypes. Homozygosity for group A *KIR* content-haplotype was observed in less than 10% of Gambians which is unusual when compared to other population groups worldwide [42].

### Potential pathological mechanisms

It is interesting that the activating *KIR3DS1* is associated with e antigen positivity and high viral loads. In addition, haplotypes carrying other activating *KIR* genes were generally linked to clearance of the surface antigen. It may be that cytolysis and inflammatory mediators generated by NK cells are important in controlling the initial infection but if unsuccessful then persistent inflammation with consequent inflammatory cell recruitment promotes conditions favourable to viral replication. The finding that activating *KIR* is linked to higher viral loads is in keeping with other studies which have linked activating *KIR* to virus related cancers [20,21] and to persistent viraemia in HCV [22], although telomeric B haplotypes are associated with slow progression of chronic hepatitis C infection in American Aboriginals [32,33].

### Implications for future research

HBV is the commonest cause of liver cancer deaths and cirrhosis deaths globally with most deaths occurring in low-income countries [1]. This is the first study to examine associations between common immune markers and liver disease in a low-income country with high hepatitis B prevalence. Future studies on a larger scale might explore these associations further providing useful mechanistic insights into pathogenesis of chronic liver disease and cancer in this context.

Whilst this investigation is the first to provide evidence that centromeric and telomeric *KIR* genotypes may be important in governing host immune responses to hepatitis B infection in Gambians, we had insufficient participants and limited genomic material to investigate the effects of HLA alleles. Participation therefore was restricted only to those for whom an adequate amount of DNA was available for KIR typing.

Additionally, in this small investigation we were unable to demonstrate the effect of functional *KIR* genes on longer term outcomes of infection such as cirrhosis and cancer. Larger studies will be necessary to provide sufficient power to examine these outcomes and may also provide the opportunity to look for effects on response to treatment for hepatitis B infection. Given the relatively large effect sizes that we observed and the lack of correlation between risk factors, hepatitis outcomes and *KIR* gene frequencies with reported ethnicity it seems unlikely that undetected population stratification would account for our findings. However this may be more of a problem in larger scale studies over wider geographical areas and it would thus be prudent to use a genomic control or structured association method to adjust for this in the analysis.

It is possible that *KIR* or *HLA* alleles and *HLA/KIR* compound genotypes will exert selective pressure on the hepatitis B virus over time in populations. In our study, viral sequences were not available to examine this hypothesis. However, such selection pressure might have important clinical effects, for example the emergence of strains carrying mutations that affect transmission or alter the configuration of proteins which are the targets of antiviral drugs. These important questions should be examined in future work which might be more effectively carried out in populations with higher frequencies of activating *KIR* genes and increased rates of homozygosity.

## Conclusions

This study evaluated the role of centromeric and telomeric *KIR* genes on outcomes of viral hepatitis infection in adult Gambians with liver disease (hepatocellular carcinoma or cirrhosis) and matched controls. We show for the first time that homozygosity for *KIR* genes belonging to the group A haplotype at the telomeric part of the KIR locus is associated with reduced risk of e antigenaemia and lower viral loads. Heterozygosity for A and B haplotypes was associated with clearance of HBsAg and control of the initial HBV infection. The mechanisms through which *KIR* genes mediate these effects warrant further assessment in larger studies.

## Supporting information

S1 Fig **Genomic organisation of (A) centromeric and (B) telomeric genotypes found in the study population.** Filled box: gene present; open box: gene absent; c-: centromeric genotype, t-: telomeric genotype, x: known or novel motifs (e.g. Bx6: sixth novel B motif identified for the first time in this study), N: number of individuals carrying the genotype of interest.
(DOC)

S1 Table KIR compound genotype distribution in the study population. c-: centromeric genotype, t-: telomeric genotype, x: known or novel motifs, N: number of individuals carrying the genotype of interest.
(DOC)
