# Supplementary material for: KIR content genotypes associate with carriage of hepatitis B surface antigen, e antigen and HBV viral load in Gambians
Source: PLoS One. 2017 Nov 17;12(11):e0188307. doi: 10.1371/journal.pone.0188307 (PMC5693433; doi:10.1371/journal.pone.0188307)
Supplement: S1 Fig — Genomic organisation of (A) centromeric and (B) telomeric genotypes found in the study population. Filled box: gene present; open box: gene absent; c-: centromeric genotype, t-: telomeric genotype, x: known or novel motifs (e.g. Bx6: sixth novel B motif identified for the first time in this study), N: number of individuals carrying the genotype of interest. (DOC) [file pone.0188307.s001.doc]

A.

| Cen genotype | N | % | ***3***  ***D***  ***L3*** | ***2***  ***D***  ***S2*** | ***2***  ***D***  ***L2*** | ***2***  ***D***  ***L3*** | ***2***  ***D***  ***L5*** | ***2***  ***D***  ***S3/S5*** | ***2***  ***D***  ***P1*** | ***2***  ***D***  ***L1*** |
| --- | --- | --- | --- | --- | --- | --- | --- | --- | --- | --- |
| c-AB2 | 68 | 24.37 |  |  |  |  |  |  |  |  |
| c-ABx1 | 43 | 15.41 |  |  |  |  |  |  |  |  |
| c-B2B2 | 28 | 10.04 |  |  |  |  |  |  |  |  |
| c-ABx4 | 25 | 8.96 |  |  |  |  |  |  |  |  |
| c-AA | 24 | 8.6 |  |  |  |  |  |  |  |  |
| c-AB1 | 15 | 5.38 |  |  |  |  |  |  |  |  |
| c-ABx7 | 14 | 5.02 |  |  |  |  |  |  |  |  |
| c-Bx16Bx17 | 11 | 3.94 |  |  |  |  |  |  |  |  |
| c-B3B3 | 9 | 3.23 |  |  |  |  |  |  |  |  |
| c-ABx5 | 8 | 2.87 |  |  |  |  |  |  |  |  |
| c-ABx3 | 7 | 2.51 |  |  |  |  |  |  |  |  |
| c-ABx8 | 6 | 2.15 |  |  |  |  |  |  |  |  |
| c-ABx2 | 3 | 1.08 |  |  |  |  |  |  |  |  |
| c-ABx6 | 3 | 1.08 |  |  |  |  |  |  |  |  |
| c-ABx10 | 2 | 0.72 |  |  |  |  |  |  |  |  |
| c-B1Bx31 | 2 | 0.72 |  |  |  |  |  |  |  |  |
| c-ABx12 | 1 | 0.36 |  |  |  |  |  |  |  |  |
| c-ABx26 | 1 | 0.36 |  |  |  |  |  |  |  |  |
| c-ABx49 | 1 | 0.36 |  |  |  |  |  |  |  |  |
| c-B1B1 | 1 | 0.36 |  |  |  |  |  |  |  |  |
| c-B1Bx15 | 1 | 0.36 |  |  |  |  |  |  |  |  |
| c-B1Bx30 | 1 | 0.36 |  |  |  |  |  |  |  |  |
| c-Bx18Bx19 | 1 | 0.36 |  |  |  |  |  |  |  |  |
| c-Bx24Bx25 | 1 | 0.36 |  |  |  |  |  |  |  |  |
| c-Bx35Bx36 | 1 | 0.36 |  |  |  |  |  |  |  |  |
| c-Bx37Bx38 | 1 | 0.36 |  |  |  |  |  |  |  |  |
| c-Bx43Bx44 | 1 | 0.36 |  |  |  |  |  |  |  |  |

B.

| Tel genotype | N | % | ***2***  ***D***  ***L4*** | ***3***  ***D***  ***L1*** | ***3***  ***D***  ***S1*** | ***2***  ***D***  ***S1*** | ***2***  ***D***  ***S4*** | ***3***  ***D***  ***L2*** |
| --- | --- | --- | --- | --- | --- | --- | --- | --- |
| t-AA | 233 | 83.51 |  |  |  |  |  |  |
| t-ABx2 | 17 | 6.09 |  |  |  |  |  |  |
| t-AB1 | 13 | 4.66 |  |  |  |  |  |  |
| t-ABx1 | 12 | 4.3 |  |  |  |  |  |  |
| t-Bx11Bx12 | 1 | 0.36 |  |  |  |  |  |  |
| t-Bx5Bx6 | 1 | 0.36 |  |  |  |  |  |  |
| t-Bx7Bx8 | 1 | 0.36 |  |  |  |  |  |  |
| t-Bx9Bx10 | 1 | 0.36 |  |  |  |  |  |  |

**S1 Fig** Genomic organisation of (A) centromeric and (B) telomeric genotypes found in the study population. Filled box: gene present; open box: gene absent; c-: centromeric genotype, t-: telomeric genotype, x: known or novel motifs (e.g. Bx6: sixth novel B motif identified for the first time in this study), N: number of individuals carrying the genotype of interest.
