# Supplementary material for: KIR content genotypes associate with carriage of hepatitis B surface antigen, e antigen and HBV viral load in Gambians
Source: PLoS One. 2017 Nov 17;12(11):e0188307. doi: 10.1371/journal.pone.0188307 (PMC5693433; doi:10.1371/journal.pone.0188307)
Supplement: S1 Table — c-: centromeric genotype, t-: telomeric genotype, x: known or novel motifs, N: number of individuals carrying the genotype of interest. (DOC) [file pone.0188307.s002.doc]

**S1 Table** KIR compound genotype distribution in the study population

| KIR profile | All | |  | Control | |  | HCC | |  | Cirrhosis | |
| --- | --- | --- | --- | --- | --- | --- | --- | --- | --- | --- | --- |
|  | N | % |  | N | % |  | N | % |  | N | % |
| c-AB2/t-AA | 46 | 16.5 |  | 26 | 18.2 |  | 12 | 12.8 |  | 8 | 19.1 |
| c-ABx1/t-AA | 43 | 15.4 |  | 27 | 18.9 |  | 11 | 11.7 |  | 5 | 11.9 |
| c-AA/t-AA | 23 | 8.2 |  | 8 | 5.6 |  | 9 | 9.6 |  | 6 | 14.3 |
| c-ABx4/t-AA | 21 | 7.5 |  | 11 | 7.7 |  | 4 | 4.3 |  | 6 | 14.3 |
| c-B2B2/t-AA | 20 | 7.2 |  | 11 | 7.7 |  | 6 | 6.4 |  | 3 | 7.1 |
| c-AB1/t-AA | 15 | 5.4 |  | 9 | 6.3 |  | 6 | 6.4 |  | 0 | 0.0 |
| c-ABx7/t-AA | 14 | 5.0 |  | 6 | 4.2 |  | 6 | 6.4 |  | 2 | 4.8 |
| c-Bx16Bx17/t-AA | 10 | 3.6 |  | 7 | 4.9 |  | 3 | 3.2 |  | 0 | 0.0 |
| c-AB2/t-ABx2 | 9 | 3.2 |  | 4 | 2.8 |  | 4 | 4.3 |  | 1 | 2.4 |
| c-ABx5/t-AA | 7 | 2.5 |  | 4 | 2.8 |  | 3 | 3.2 |  | 0 | 0.0 |
| c-B3B3/t-AA | 7 | 2.5 |  | 3 | 2.1 |  | 4 | 4.3 |  | 0 | 0.0 |
| c-AB2/t-AB1 | 6 | 2.2 |  | 2 | 1.4 |  | 3 | 3.2 |  | 1 | 2.4 |
| c-AB2/t-ABx1 | 6 | 2.2 |  | 3 | 2.1 |  | 2 | 2.1 |  | 1 | 2.4 |
| c-ABx3/t-AA | 6 | 2.2 |  | 3 | 2.1 |  | 3 | 3.2 |  | 0 | 0.0 |
| c-ABx8/t-AA | 5 | 1.8 |  | 1 | 0.7 |  | 4 | 4.3 |  | 0 | 0.0 |
| c-ABx2/t-AA | 3 | 1.1 |  | 0 | 0.0 |  | 1 | 1.1 |  | 2 | 4.8 |
| c-ABx6/t-AA | 3 | 1.1 |  | 3 | 2.1 |  | 0 | 0.0 |  | 0 | 0.0 |
| c-B2B2/t-AB1 | 3 | 1.1 |  | 1 | 0.7 |  | 1 | 1.1 |  | 1 | 2.4 |
| c-B2B2/t-ABx2 | 3 | 1.1 |  | 1 | 0.7 |  | 1 | 1.1 |  | 1 | 2.4 |
| c-ABx4/t-ABx2 | 2 | 0.7 |  | 1 | 0.7 |  | 1 | 1.1 |  | 0 | 0.0 |
| c-B1Bx31/t-AA | 2 | 0.7 |  | 1 | 0.7 |  | 1 | 1.1 |  | 0 | 0.0 |
| c-B2B2/t-ABx1 | 2 | 0.7 |  | 0 | 0.0 |  | 1 | 1.1 |  | 1 | 2.4 |
| c-AA/t-ABx1 | 1 | 0.4 |  | 1 | 0.7 |  | 0 | 0.0 |  | 0 | 0.0 |
| c-AB2/t-Bx7Bx8 | 1 | 0.4 |  | 1 | 0.7 |  | 0 | 0.0 |  | 0 | 0.0 |
| c-ABx10/t-AA | 1 | 0.4 |  | 0 | 0.0 |  | 1 | 1.1 |  | 0 | 0.0 |
| c-ABx10/t-AB1 | 1 | 0.4 |  | 1 | 0.7 |  | 0 | 0.0 |  | 0 | 0.0 |
| c-ABx12/t-ABx2 | 1 | 0.4 |  | 1 | 0.7 |  | 0 | 0.0 |  | 0 | 0.0 |
| c-ABx26/t-AA | 1 | 0.4 |  | 0 | 0.0 |  | 1 | 1.1 |  | 0 | 0.0 |
| c-ABx3/t-AB1 | 1 | 0.4 |  | 0 | 0.0 |  | 1 | 1.1 |  | 0 | 0.0 |
| c-ABx4/t-AB1 | 1 | 0.4 |  | 0 | 0.0 |  | 1 | 1.1 |  | 0 | 0.0 |
| c-ABx4/t-ABx1 | 1 | 0.4 |  | 0 | 0.0 |  | 0 | 0.0 |  | 1 | 2.4 |
| c-ABx49/t-AA | 1 | 0.4 |  | 1 | 0.7 |  | 0 | 0.0 |  | 0 | 0.0 |
| c-ABx5/t-ABx2 | 1 | 0.4 |  | 1 | 0.7 |  | 0 | 0.0 |  | 0 | 0.0 |
| c-ABx8/t-ABx2 | 1 | 0.4 |  | 1 | 0.7 |  | 0 | 0.0 |  | 0 | 0.0 |
| c-B1B1/t-Bx9Bx10 | 1 | 0.4 |  | 0 | 0.0 |  | 0 | 0.0 |  | 1 | 2.4 |
| c-B1Bx15/t-AA | 1 | 0.4 |  | 0 | 0.0 |  | 1 | 1.1 |  | 0 | 0.0 |
| c-B1Bx30/t-AA | 1 | 0.4 |  | 1 | 0.7 |  | 0 | 0.0 |  | 0 | 0.0 |
| c-B3B3/t-AB1 | 1 | 0.4 |  | 1 | 0.7 |  | 0 | 0.0 |  | 0 | 0.0 |
| c-B3B3/t-ABx1 | 1 | 0.4 |  | 1 | 0.7 |  | 0 | 0.0 |  | 0 | 0.0 |
| c-Bx16Bx17/t-ABx1 | 1 | 0.4 |  | 0 | 0.0 |  | 0 | 0.0 |  | 1 | 2.4 |
| c-Bx18Bx19/t-AA | 1 | 0.4 |  | 1 | 0.7 |  | 0 | 0.0 |  | 0 | 0.0 |
| c-Bx24Bx25/t-AA | 1 | 0.4 |  | 0 | 0.0 |  | 0 | 0.0 |  | 1 | 2.4 |
| c-Bx35Bx36/t-Bx5Bx6 | 1 | 0.4 |  | 0 | 0.0 |  | 1 | 1.1 |  | 0 | 0.0 |
| c-Bx37Bx38/t-AA | 1 | 0.4 |  | 0 | 0.0 |  | 1 | 1.1 |  | 0 | 0.0 |
| c-Bx43Bx44/t-Bx11Bx12 | 1 | 0.4 |  | 0 | 0.0 |  | 1 | 1.1 |  | 0 | 0.0 |

c-: centromeric genotype, t-: telomeric genotype, , x: known or novel motifs, N: number of individuals carrying the genotype of interest.
